# Renal Cell Carcinoma with Duodenal Metastasis: Is There a Place for Surgery? A Review

**DOI:** 10.3390/jcm14207189

**Published:** 2025-10-12

**Authors:** Fayek Taha, Rami Rhaiem, Stephane Larre, Ali Reza Kianmanesh, Yohan Renard, Belkacem Acidi

**Affiliations:** 1 Département d’Urologie, Centre Hospitalo-Universitaire de Reims, 45 rue Cognac Jay, 51092 Reims Cedex, France; 2 Département de Chirurgie Viscérale et Endocrinienne, Centre Hospitalo-Universitaire de Reims, 45 rue Cognac Jay, 51092 Reims Cedex, France; rrhaiem@chu-reims.fr (R.R.); yrenard@chu-reims.fr (Y.R.); 3 Département de Chirurgie Oncologique, Institut Gustave Roussy, 114 Avenue Paul Vaillant Couturier, 94804 Villejuif Cedex, France; 4 INSERM, Unit 1193, 94800 Villejuif, France

**Keywords:** renal cell carcinoma, duodenal metastasis, metastatic disease

## Abstract

**Introduction**: Renal cell carcinoma (RCC) develops metastatic disease in 30–50% of patients during their disease course, with approximately one quarter presenting with metastases at diagnosis. While the lungs, liver, bones, brain, and adrenal glands are the most frequent metastatic sites, duodenal involvement is exceptionally rare. This uncommon presentation poses diagnostic and therapeutic challenges, particularly regarding the role of surgical resection in the metastatic setting. **Objective**: We aim to evaluate the clinical presentation, management strategies, and outcomes of patients with duodenal metastasis from RCC, with particular emphasis on the potential role of surgery, through a systematic review of the literature. **Methods**: A comprehensive electronic search of Medline, Embase, and Scopus was conducted according to PRISMA guidelines. The following MeSH terms were applied: Kidney Neoplasms [MeSH] AND Duodenal Neoplasms/metastasis [MeSH]. Eligible studies included original reports or case series describing RCC with duodenal metastasis. Demographic, clinical, surgical, and survival data were extracted and synthesized. **Results**: Of 89 records identified, 83 underwent full-text review and 51 met inclusion criteria, representing 55 patients. The median age at diagnosis was 64 years, and 80% of primary tumors arose from the right kidney. Nearly all patients (98%) were symptomatic, most commonly with upper gastrointestinal bleeding, anemia, or obstructive features. Pancreaticoduodenectomy was the predominant surgical approach, performed with curative intent in selected cases. Patients undergoing surgery achieved a 5-year overall survival of 70%, compared with 0% among non-operated patients. **Conclusions**: Duodenal metastasis from RCC remains an uncommon entity, limiting the strength of available evidence. Nevertheless, our findings suggest that surgical management—when feasible and decided within a multidisciplinary framework—can provide meaningful survival benefit and should be considered as a complement to contemporary systemic therapies for metastatic RCC

## 1. Introduction

Renal cell carcinoma (RCC) represents approximately 2–3% of all adult malignancies and is characterized by a wide spectrum of clinical behavior. Despite advances in imaging and earlier detection, 30–50% of patients develop metastatic disease during their disease course. More than 20% of patients will develop metastasis after curative-intent nephrectomy, underscoring the systemic nature of the disease even when initially localized. In addition, 20–30% of cases are diagnosed de novo at a metastatic stage [1].

According to French and European guidelines, surgical resection of metastases (metastasectomy) should always be considered when technically and oncologically feasible, as it has been associated with improvement in overall survival, particularly in the setting of oligometastatic disease [1,2]. This approach is supported by retrospective series and selected prospective data, which suggest that complete resection of limited metastatic deposits may offer prolonged disease control and, in some cases, durable remission.

The most frequent metastatic sites for RCC include the lung, liver, bone, brain, and adrenal gland. Interestingly, RCC exhibits unique metastatic patterns compared with other solid tumors, including a well-documented tropism for the pancreas. RCC has been cited as the most common primary tumor leading to solitary pancreatic metastasis, whereas duodenal metastasis remains distinctly uncommon. Autopsy series indicate that RCC accounts for approximately 7% of all metastatic lesions identified in the small bowel [3], suggesting that the true incidence may be underestimated during life due to the often nonspecific or silent presentation of such lesions.

Duodenal metastases, when encountered, most frequently originate from melanoma, lung cancer, breast cancer, or thyroid cancer [4,5], Metastatic spread from RCC to the duodenum is therefore unusual and may present diagnostic and therapeutic challenges, particularly because symptoms—when present—tend to be nonspecific, including anemia, gastrointestinal bleeding, or obstructive phenomena. While sporadic case reports have described duodenal involvement by RCC, the literature remains fragmented, and, to date,, no systematic review has been recently published on this specific metastatic site.

Here, we systematically compile and analyze all published cases of duodenal metastasis from RCC to describe patient and tumor characteristics, evaluate management strategies and outcomes, and assess the specific role and potential benefits of surgical intervention.

## 2. Methods

The PRISMA (Preferred Reporting Items for Systematic Reviews and Meta-Analyses) Statement guidelines were followed throughout the entire process to ensure methodological transparency and reproducibility [6]. A comprehensive electronic search was carried out to identify all published studies from January 1995 to January 2021. The Medline, Embase, and Scopus databases were selected as they represent the most widely used and complementary biomedical sources, covering both clinical and surgical literature. The following combination of keywords was used to identify relevant studies: (“Kidney Metastasis” OR “Carcinoma, renal cell”) AND (“duodenum” OR “duodenum metastasis”). In addition to database searching, the reference lists of all selected papers were manually screened to identify potential additional studies not captured during the initial search strategy.

Studies were included if they contained original data on patients diagnosed with duodenal metastasis from RCC. Review articles without original patient data, editorials, and duplicated data across publications were excluded. Only articles published in English were selected for analysis to ensure accurate interpretation of clinical and surgical details.

The screening process was performed in two distinct stages. First, titles and abstracts were independently reviewed by two trained reviewers to assess preliminary eligibility. Any disagreement at this stage was resolved through discussion with two senior reviewers. Second, the full-text versions of all shortlisted studies were obtained and assessed for final inclusion or exclusion by two independent researchers using the same consensus process. Data extraction was then performed independently by the reviewers, and any discrepancies were resolved by agreement or by referral to the two senior authors. In cases where data were incomplete or missing, attempts were made to contact the corresponding author (when contact information was available in the article) to obtain additional details.

From each eligible study, the following data were collected:

(1) Patient characteristics: age, sex.
(2) Clinical, biological, and radiological presentation: symptoms at diagnosis, biological abnormalities, CT scan results, endoscopic features, and other metastatic sites.
(3) Tumor characteristics: side of the primary renal cancer, synchronous or metachronous presentation, and disease-free interval (defined as the time between resection of the initial tumor and diagnosis of duodenal metastasis).
(4) Treatment and perioperative course: type of surgery performed and reported complications.
(5) Outcome: overall survival.

For statistical analysis, patients were classified into two groups: those who underwent surgery and those managed without surgical resection. Comparisons between groups were performed using the Mann–Whitney test for continuous variables and Fisher’s exact test or Chi-square test for categorical variables. Survival analyses were performed using the Kaplan–Meier method with available outcome data on overall survival, and survival curves were compared using the log-rank test.

## 3. Results

### 3.1. Data Extraction

**Figure 1:**
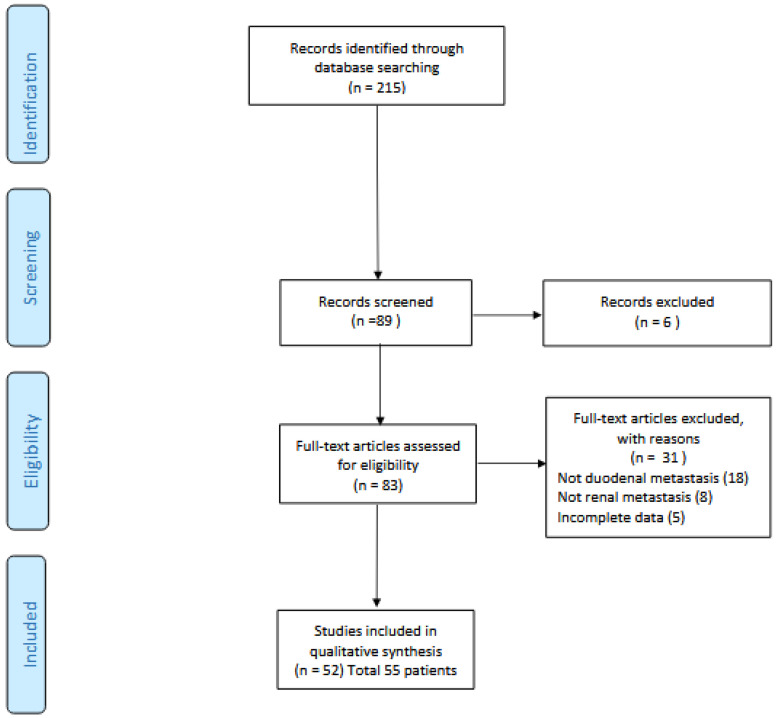
Flow chart.

A total of 89 articles were initially identified through the database search. After removing duplicates and applying the initial screening process, 83 full-text articles were assessed for eligibility. Following the application of predefined inclusion and exclusion criteria, 51 articles were ultimately included, representing 55 individual patients diagnosed with duodenal metastasis from RCC (Figure 1: flow chart).

The rarity of eligible cases illustrates the uncommon nature of this condition and explains why most available evidence is limited to isolated case reports or small series. To ensure transparency, all patient-level data extracted from the included studies are detailed in Table 1. For clarity and synthesis, overall cohort characteristics were pooled and summarized in Table 2, which reports mean values and proportions together with their 95% confidence intervals.

### 3.2. Haracteristics of Patients

The mean age at diagnosis of duodenal metastasis was 64 years, and the sex ratio was markedly in favor of males, with a male-to-female ratio of 4:1. Most of the metastases were metachronous, occurring in 49 patients after an initial disease-free interval. Among patients with synchronous metastases, the average disease-free interval (DFI) was 7 years, ranging widely from as short as 8 months to as long as 33 years after the primary nephrectomy.

Concomitant metastatic sites were present in 20 patients (36%), underlining the systemic nature of advanced RCC in a significant proportion of cases. Regarding the primary tumor, the right kidney was identified as the origin in 28 patients (58%). The duodenal metastatic site most frequently involved was the second portion of the duo-denum, which was affected in 35 patients (80%). Within this subgroup, 12 patients (27%) had a peripapillary localization, a finding with potential implications for biliary and pancreatic duct involvement.

### 3.3. Clinical Presentation

A wide spectrum of clinical presentations was observed, as detailed in Table 2. Overall, duodenal metastases were symptomatic in 54 of the 55 patients (98%), indicating that incidental detection was exceptional. The most frequent initial presentation was gastrointestinal bleeding, which was reported in 36 patients (65%). This bleeding was either manifested as upper gastrointestinal hemorrhage (12 patients, 22%) or as melena and/or hematochezia (26 patients, 47%). Anemia, often secondary to chronic blood loss, was documented in 32 patients (58%). These findings emphasize that digestive bleeding and related anemia are the dominant modes of presentation in this rare metastatic setting.

### 3.4. Complementary Exams

A CT scan was performed in 39 patients (71%), providing the first morphological assessment of duodenal involvement. Among these, 27 patients (69%) demonstrated either a duodenal mass or focal wall thickening, typically with the classical radiological appearance of a hypervascularized lesion. Biliary duct dilatation was reported in 6 cases (15%), reflecting the potential for secondary obstruction. Notably, in 5 patients (12%), CT failed to reveal any abnormality in the duodenum or pancreas, underlining the possibility of false-negative results and the limits of cross-sectional imaging in this context.

Endoscopic examination was conducted in 51 patients (91%) and offered complementary diagnostic information. The most frequent finding was a submucosal mass, often ulcerated or actively bleeding, consistent with the vascular nature of RCC metastases. In a minority of patients (8%), the only abnormality was a solitary ulcer without an obvious mass, emphasizing the heterogeneity of mucosal involvement and the importance of obtaining biopsies in any suspicious lesion in patients with a prior history of RCC

## 4. Treatment and Outcome

### 4.1. Treatment Strategy

Therapeutic management was reported in 48 patients. Surgery alone was performed in 23 patients (48%), while 5 patients (10%) received a combination of surgery and systemic therapy. Eleven patients (23%) were managed with medical treatment alone, the most frequent regimen being antiangiogenic therapy, which was used in 61% of cases receiving systemic treatment.

In the specific context of non-controlled tumor bleeding, different approaches were adopted. One patient underwent urgent surgical pancreaticoduodenectomy, while another was treated with endoscopic thermocoagulation. However, the most commonly used hemostatic intervention was radiological embolization, performed in 3 patients.

In case of non-controlled tumor bleeding: a surgical treatment with pancreaticoduodenoctomy was performed in one patient, and endoscopic thermocoagulation in one other patient. But the most used hemostatic procedure was radiological embolization performed in 3 patients.

### 4.2. Surgical Procedure Outcomes and Comparison with Non Surgical Procedure Management

Among surgical patients, the most frequent procedure was pancreaticoduodenectomy, performed in 20 cases (76%). Other surgical approaches included wedge resection or more limited procedures such as ampullectomy in 6 patients (24%). Early postoperative outcomes were reported in 23 patients. Major complications (Clavien–Dindo grade ≥ III) occurred in three cases: two patients developed an anastomotic leak, and one patient died on postoperative day 40 from sepsis or bleeding. Additionally, two patients experienced postoperative gastroplegia. Of the operated cohort, 7 patients (26%) were noted to have other suspected metastatic sites at the time of surgery, and in two cases local treatment of these additional sites was undertaken simultaneously (Table 3).

**Figure 2:**
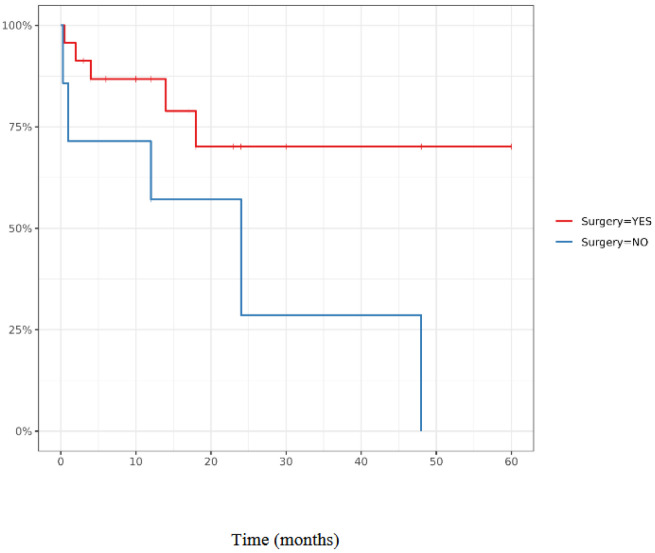
Overall Survival.

When comparing surgical to non-surgical management, operated patients showed markedly improved survival (Figure 2). The 5-year overall survival rate reached 70% in the surgical cohort, whereas no long-term survivors were reported among patients managed without surgery. While these results highlight the potential benefit of resection, they must be interpreted cautiously given the high risk of selection bias and the limited number of cases.

## 5. Discussion

### 5.1. Features of Duodenal Metastases

The dissemination of renal cell carcinoma (RCC) to the duodenum can occur through several mechanisms, including hematogenous spread, lymphatic dissemination, transcoelomic seeding, or direct invasion from the primary tumor. Among these, direct invasion appears to be the most frequent pathway, a finding that can be largely attributed to the close anatomical relationship between the right kidney and the duodenum. This observation is consistent with the predominance of right-sided primaries reported in the included studies, suggesting that anatomical proximity plays a central role in this metastatic pattern.

Nevertheless, the presence of metachronous lesions, sometimes occurring several years after nephrectomy, indicates that hematogenous dissemination may also be a relevant mechanism in selected cases. This highlights the biological heterogeneity of RCC and its well-known ability to generate late and unusual metastatic deposits.

In metachronous presentations, an interesting but unanswered question is whether the status of surgical margins following nephrectomy could be associated with the later occurrence of duodenal metastases. Unfortunately, none of the articles reviewed provided details on margin status, preventing further exploration of this hypothesis.

From a clinical standpoint, symptoms were present in 98% of patients, and in most cases these were related to gastrointestinal bleeding. Presentations ranged from occult bleeding with anemia to massive hemorrhage requiring emergency hemostatic intervention. The high incidence of bleeding can be explained by the typical hypervascularization of renal cell carcinoma lesions.

From a diagnostic perspective, CT scans failed to show any abnormality in 12% of cases, highlighting the crucial role of endoscopic examination. While the endoscopic presentation most frequently consists of a mass or polypoid lesion, in some patients only an ulcer is observed. This underlines the importance of performing biopsies in any suspicious lesion in patients with a prior history of cancer. The case report by Chara et al. [26] illustrates the consequences of a missed opportunity: an ulcer was not biopsied, leading to a delayed diagnosis.

### 5.2. Role of Metastasectomy in Oligometastatic Renal Cell Carcinoma

Up to now, it is considered that any patient with solitary metastatic RCC should be a candidate for a complete surgical excision if medical and technically possible according to European and French guidelines [1] even if no prospective randomized clinical trials have been realized so far. Indeed, those recommendations are based on retrospectives studies. We can notice that the utilization of metastasectomy increased from 24.9% in 2006 to 31,4% in 2013 [57].

In the review of Dabestani et al. [58], complete metastasectomy was associated with significantly longer survival rates in median of medians (OS or CSS) 40.8 [31.6–48.0] vs. 14.8 months [13.2–21 months] compared with incomplete or no metastasectomy.

Candidate patients for metastasis resection must be selected according to specific criteria, according to the multiple reviews [59,60,61]: Age, performance status, favorable MSKCC, high DFI, low metastatic volume.

In metastasectomy of RCC, intraoperative complications and major complications (Clavien III–IV) were found in 7.9 and 25.1% of patients, respectively, with in-hospital mortality rate of 2.4% [62].

Pancreas metastases are similar to duodenal metastases in some points, especially for surgery technique. A recent meta-analysis of 354 patients with pancreatic metastases showed a 5-year overall survival of 53.9% for patients who had surgical resection of pancreatic tumors secondary to RCC [63]. Pancreaticoduodenectomy was performed in 119 patients.

In their review, Hall et al., defined criteria for patients who should be candidate for surgery in case of pancreatic metastases [59]:

- Asymptomatic presentation
- No extrahepatic diseases
- Solitary metastasis
- Absence of vascular invasion
- Ability to complete resect tumor

### 5.3. Duodenal Metastases of RCC: Surgery and Outcome

Our review, which includes all published cases over the last 25 years, suggests that patients undergoing surgical resection have a better survival prognosis. However, the data remain insufficient to draw firm conclusions, mainly because of several biases, including publication bias and selection bias in survival analyses. In contrast to pancreatic metastases, which are symptomatic in only 45% of patients [64] duodenal metastases are symptomatic in nearly all reported cases. The standard surgical approach for duodenal metastases is classical pancreaticoduodenectomy, although wedge or partial resections may be performed in selected cases. This differs from pancreatic metastases, where pancreaticoduodenectomy is less frequently performed and other, more limited resections are often possible [27].

Unlike pancreatic metastases from RCC, which are associated with comparatively favorable survival outcomes, no similar prognostic advantage has been demonstrated for duodenal metastases, underlining the distinct and less favorable nature of this metastatic site.

Of course, when metastatic sites are multiples and diffused, introduction of targeted therapy must be indicated. Moreover, currently we have a lot of new drugs available for treatment of metastatic renal cancer with proved efficiency, and arrival of immunotherapy checkpoint inhibitors has changed the medical strategy in medical treatment of metastatic RCC [1]. One potential advantage of metastasectomy, when feasible, is the possibility of delaying systemic therapy, thereby postponing the exposure to drug-related toxicities.However, the interpretation of these findings must take into account several limitations. Beyond publication and selection biases, data on disease-free survival (DFS), were inconsistently reported across case reports. This heterogeneity precluded any reliable pooled analysis and represents an additional limitation of the present review.

A major limitation of the present review is the inherent risk of publication bias, since positive outcomes are more likely to be reported, and selection bias, as patients offered surgery are usually younger, fitter, and with more favorable disease characteristics. These factors must be taken into account when interpreting the apparent survival advantage associated with surgical management.

When resection is not possible for a symptomatic tumor, palliative treatments may be appropriate. Some authors have reported successful embolization of RCC duodenal metastases to control persistent bleeding. For biliary obstruction, endoscopic placement of a stent may be indicated [65].

Finally, a few cases have also been treated with radiotherapy, though the evidence remains anecdotal, and its role is not well established in this context.

## 6. Conclusions

The clinical features of duodenal metastases from renal cell carcinoma (RCC) are largely similar to those of primary duodenal tumors, with the notable exception of a higher propensity for significant bleeding, likely due to the marked hypervascularization characteristic of RCC. In nearly all reported cases, duodenal metastases are symptomatic at the time of diagnosis.

Imaging and endoscopy are useful diagnostic tools, but histological confirmation remains mandatory. Immunohistochemistry plays a crucial role in establishing the renal origin of the lesion and distinguishing it from other primary or secondary tumors.

Although the number of reported cases is low, and most publications include only one or two patients, the available evidence suggests that duodenal metastasis represents a poor prognostic location, mainly due to its local complications. The rarity of this condition precludes large-scale series, introducing potential biases such as publication and selection bias that may overestimate the apparent benefit of surgery.

By analogy with other oligometastatic RCC sites, surgical resection should be considered within a multidisciplinary tumor board, but decisions must take into account patient-specific factors and the feasibility of complete resection. For non-resectable but symptomatic tumors, palliative approaches such as embolization or stenting may provide effective symptom control.

Finally, systemic therapy remains a cornerstone of management in metastatic RCC. The rapid evolution of targeted agents and immune checkpoint inhibitors is reshaping treatment paradigms and will likely redefine the role of metastasectomy in carefully selected patients over the coming decade.

## Figures and Tables

**Table 1:** Summary of all published cases of duodenal metastases of Renal Cell Carcinoma.

Ref.	Gender, Age	Clinical Presentation	Biological Abnormalities	CT SCAN	Endoscpîc Aspect/Localisation	Histological Proof of Renal Carcinoma	Nephrectomy Side	Delay After Nephrectomy	Treatment	Patient Outcome	Other Localization	Operated Patients- Complications
Black, 1995 [7]	F, 60 years	Melena	Severe anemia	Mass invading pancreas	Periampullary lesion	Surgical sample	Right	Metachronous-6 years	Surgery		-	
Hsu, 1996 [8]	F, 54 years	Melena, anemia	Anemia	-	Second duodenum-polypoid mass	Endoscopic biopsy	-	Metachronous-1.5 years	Chemotherapy	Dead at 1 month	-	
Hsu, 1996 [8]	F, 72 years	Melena, anemia	Anemia	Localized right renal tumor	Second duodenum-Submucosal mass with ulcerations	Endoscopic biopsy	Left	Metachronous-1 year	Treatment refused	Lost to follow up	Bone	
Leslie, 1996 [9]	M, 53 years	Melena, weight loss	Bilirubin increase		Periampullary lesion	Endoscopic biopsy	Right	Metachronous 8 years	PD	Alive at 2.5 years	-	No complication
Toh, 1998 [10]	F, 59 years	Lethargy, abdominal pain, anorexia, weight loss, iron suplementation for chronic anemia	Anemia	Solid mass on the posterior wall of Duodenum	Normal	Surgical sample	Right	Metachronous-10 years	Wedge resection	-	-	Gastroplegia
Janzen, 1998 [11]	M, 75 years	Melena, bleeding necessitating ressucitation	Severe anemia	Perimapullary mass extending to head of the pancreas	Periampullary mass	Endoscopic biopsy	Left	Metachronous-18 years			-	
Yavascaoglu, 1999 [12]	M, 63 years	Right flank pain, melena	-	Right renal tumor of 15 × 14 cm and pancreatico-duodenal mass 7 × 5 cm	Hemorrhagic area on the second duodenum	Endoscopic biopsy	Right	Synchronous	PD + interferon-Nephrectomy	Alive at 23 months	-	-
Ohmura, 2000 [13]	M, 62 years	Occlusion		Enhanced mass of renal fossa invading psoas muscle and duodenum	Large elvated irregular shaped tumor, obstructive	Surgical sample	Left	Metachronous-5 years	Wedge resection	Dead at 1 month	Lung (surgery)	Peritonitis/bleeding at day-40: death
Hashimoto, 2001 [14]	M, 68 years	Dyspnea	Severe anemia	Wall thickening	Ulcerated tumor on duodenal bulb	Surgical sample	Left	Metachronous-11 years	PD	Alive at 18 months	-	No complication
Lee, 2002 [15]	F, 76 years	Dyspepsia, abdominal pain, lump on upper right abdomen	-	Wall thickening		Endoscopic biopsy	Left	Metachronous-4 years	Medical treatment	Alive at 1 year	-	
Loualidi, 2004 [16]	M, 76, years	Weakness, dizziness, dyspnea, epigastric discomfort	Anemia	-	Periampullary–lobular mass	Endoscopic biopsy	Right	Metachronous-5 years	Paliative radiotherapy	Alive at 1 year	L5	
Chang, 2004 [17]	F, 63 years	4 episodes of bleeding in 9 months	Severe anemia	-	Duodenal bulb-ulcerative mass	Endoscopic biopsy	Right	Metachronous-9 years	Surgery	Alive at 10 months	-	No complication
Bhatia, 2006 [18]	M, 55 years	Jaundice, abdominal lump	Cytolysis, cholestasis	No mass	Second duodenum-submucosal lesion	Endoscopic biopsy	Left	Metachronous-1 year	Paliative radiotherapy	Lost to follow up	Liver	
Sadler, 2007 [19]	M, 67 years	Melaena, lethargyy, recurent anemia ++	Severe anemia	Polypoid mass, in the medial wall of D2 arising from pancreas & renal mass	Angulus-polyp	Endoscopic biopsy	Left	Synchronous	Nephrectomy + interferon	Dead 2 years	Lung & adrenal gland left	
Sadler, 2007 [19]	M, 75 years	Chronic anemia	-	Pancreatic mass invading duodenum	Duodenal bulb-polyp	Endoscopic biopsy	Right	Metachronous-9 years	No treatment	-	-	
Adamo, 2008 [20]	M, 86 years	Obstruction & anemia	-	Duodenal mass invading pancreas	-	Surgical sample	-	Metachronous-13 years	PD	Alive at 7 months	-	Pulmonary embolism
Das, 2009 [21]	M, 68 years	Hematochezia	Anemia	-	Second duodenum-large friable, centrally ulcerated mass	Endoscopic biopsy	-	Metachronous-10 years	PD	Alive at 4 months	Lung	
Teo, 2010 [22]	M, 50 years	Jaundice, abdominal pain	Cholestasis, cytolysis, bilirubin increase	Dilatation of the biliary duct	periampullary-mass	Endoscopic biopsy	Left	Metachronous-3 years	Antiangiogenics	1 week	Lung	
Kanthan, 2010 [3]	M, 69 years	Upper gastrointestinal bleeding	-	-	-	Endoscopic biopsy	-	Metachronous-8 years	-	-	-	
HE, 2010 [23]	M, 62 years	Massive melena	Anemia, elevated CA 19-9 and ACE	Submucous tumor	Bleeding mass second duodenum	Surgical sample	Right	Metachronous-11 years	PD	Alive at 17 months	-	Gastroplegia
Rustagi, 2011 [24]	M, 66 years	Melena, anemia, fatigue	Severe anemia	Mass	Second duodenum-actively bleeding and ulcerative mass	Endoscopic biopsy	Bilateral	Metachronous-13 years	PD	Dead at 2 weeks	-	Leakage of pancreaticojejunostomy fistula-sepsis
Cherian, 2011 [25]	M, 80 years	Syncope, melena	Severe anemia	Soft tissue mass extending in duodenum from right nephrectomy bed	Second duodenum	Endoscopic biopsy	Right	Metachronous-1 years	Sorafenib, everolimus	Dead at 1 year	Lumbar vertebra-lung	
Chara, 2011 [26]	M, 49 years	Melena		Enlargement of the head of the pancreas	Duodenal ulcer initially treated by sclerotherapy and biopsied 5 months after diagnosis	Surgical sample	Left	Metachronous-6 years	PD	Alive at 4 years	-	No complication
Yang, 2012 [27]	F, 72 years	Melena, fatigue, hematemesis	Severe anemia	-	Mass at second portion of duodenum bleeding and necrosis	Surgical sample	Left	Metachronous-10 years	Wedge resection	Recurrence at 10 months	-	Surgical site infection-Pulmonary infection
Mandal, 2012 [28]	F, 60 yars	Melena-diarrhea	Nothing	No lesion on CT Scan	Second duodenum-abnormal duodenal border	Endoscopic biopsy	Left	Synchronous	Nephrectomy + chemotherapy	-	Skin	
Zhao, 2012 [29]	M, 56 years	Occlusion-bleeding-fatigue	Severe anemia	filling defect	Second duodenum-ulcerated and hemorrhagic mass	Endoscopic biopsy	Right	Metachronous-7 years	Wedge resection	Alive at 1.5 years	Tail pancreatic lump non operated	No complication
Chen, 2012 [30]	F, 76 years	Hematoschzia, anemia	Anemia	Hypervascular lesion involving duodenum, pancreas gastric antrum	First duodenum-submucosal mass involving stomach	Surgical sample	Left	Metachronous-6 years	PD	Aliver at 24 months	-	No complication
Schlussel, 2012 [31]	M, 53 years	Melana, fatigue,	Severe anemia	Large mass (15 cm) arising of right kidney protruding into duodenum lumen	Second duodenum -bleeding mass	Surgical sample	Right	Synchronous	Nephrectomy + PD + thrombectomy + IL-2	Alive at 3 months	Lung	No complication
Gonzalo-Marin, 2012 [32]	M, 66 years	Melena	Anemia	Necrotic right renal mass with duodenum invasion-bile duct dilatation	Second duodenum-large and deep ulceration with edematous borders	Endoscopic biopsy	Right	Metachronous	-	-	Paraaortic LN-lUng	-
Vashi, 2012 [33]	M, 53 years	Dyspnea	Severe anemia	Right renal lesion	First duodenum-2 small vascular polyps	Endoscopic biopsy	Right	Metachronous	Duodenotomy		Small intestine polyps	No complication
Ashraf Teli, 2012 [34]	M, 52 years	Gastro intestinal bleeding		Duodenal metastasis	Duodenal mass, bleeding	Surgical sample	Right	Metachronous-8 years	Duodenotomy	Died at 4 months	-	No complication
Karakatsanis, 2013 [35]	M, 77 years	Isolated jaundice	-	Dilatation of the biliary duct	Ampula-massI25	Endoscopic biopsy	Right	Metachronous-3 years	Ampullectomy + biliary stent	Dead at 1.5 year	Lung/bone	
Hata, 2013 [36]	M, 50 years	Anemia, melena	Severe anemia		Second duodenum-Ulcerated polypoid mass (not involving ampulla); NBIde,se assembly of microvessels	Endoscopic biopsy	Left	Metachronous-15 years	PD	Alive at 1 year	Bone (radiotherapy)-pancreas tail (surgery)	Leakage of gastrojejunostomy
Neofytou, 2014 [37]	M, 41 years	Massive GI bleeding	Anemia	-	Vater ampula	Endoscopic biopsy	Left	Metachronous-1 year	Initially under pazopanib, PD for emergency	Dead at 2 months	Adrenal gland bilatera, thoracic lymphnodes	
Espinoza,2014 [5]	M, 58 years	Gastro intestinal bleeding	-	-	-	-	Right	Metachronous-12 years	PD	Alive at 12 months	-	
Espinoza,2014 [5]	F, 66 years	Abdominal pain	-	-	-	-	Right	Metachronous-4 years	PD	Alive at 12 months	-	
Haidong, 2014 [38]	M, 50 years	Fatigue–fever– diarrhea	Bilirubin increase-Cholestasis- moderate cytolysis	Presence of tumor	No endoscopy	Surgical sample	Right	Synchronous	PD + Radical nephrectomy FN alph-2b	Alive at 5 years	-	No complication
Hu, 2014 [39]	M, 57 years	Dyspnea, dyspepsia, fatigue, anemia, melena	Severe anemia	Intraluminal enhancing lesion 6 cm	Bulb/second duodenum-polypoid ulcerative and friable mass bleeding with obstruction	Endoscopic biopsy	Right	Metachronous-12 years	PD + sunitinib	Alive at 6 months	-	No complication
Osama Mohamed, 2015 [40]	M, 80 years	Melena, abdominal pain, dyspnea	Anemia	8 cm mass lower pole of kidney invading duodenum	polypoidal mass anterior second duodenum bleeding	-	Right	Synchronous	Palliative treatment	-	-	
Vootla, 2015 [41]	M, 74 years	Melena, weight loss	Anemia		Duodenal nodule	Endoscopic biopsy	Left	Metachronous-4 years	Paliative radiotherapy	-	Oesophagus	
Geramizadeh, 2015 [42]	M, 61 years	Melena	Normal	Mass invading duodenum and pancreas	Second duodenum-ulcer aspect	Endoscopic biopsy	Right	Metachronous-16 years	PD		-	No complication
Jideh, 2016 [43]	M, 50 years	Fever-abdominal pain-jaundice	Bilirubin increase-cholestasis	Biliary duct dilatation-no mass shown	Periampullary-mucosal mass	Endoscopic biopsy	Right	Metachronous	-	-	-	
Sarocchi, 2016 [44]	M, 57 years	GI bleeding			Vater ampula irregular	Endoscopic biopsy	Left	Metachronous-3.5 years	PD	Alive a 4 years	-	No complication
Saito, 2017 [45]	M, 64 years	Abdominal pain, fever, anorexia, weight loss	-	Hypervaslucar lesion duodenum and pancreatic head	Second duodenum apart form apula-submucosal mass with central ulcer	Endoscopic biopsy	Left	Metachronous-25 years	Pazopanib	-	Concomitant lymphoma treated-lung	
Omranipour, 2017 [46]	M, 59 years	Melena	Anemia	Mass in nephrectomy bed invading D2 and D3	Irregular, polypoid, ulcerative mass in second portion of duodenum	Endoscopic biopsy	Right	Metachronous-6 years	PD	Alive a 1 year	-	No complication
Villela-Segura, 2018 [47]	F, 48 years	Burning and sharp epigastric pain, hematemesis, melena, fever, hypotension	Severe anemia	Mass in the second portion of duodenum	Mass at second portion of duodenum, submucosal, ulcerated surface, bleeding, 90% of stenosis	Endoscopic biopsy	-	Metachronous-1 year	-	Dead at 1 week	-	
Podboy, 2018 [48]	M, 80 years	Melena	-	Hypervascularized mass of pancreas and duodenum	Ulcerated mass	Endoscopic biopsy	-	Metachronous-15 years	-	-	-	
Ignatavicius, 2018 [49]	M, 62 years	Severe upper abdominal pain, jaundice, lethargy, subclinical obstruction	Bilirubin increase	Periampummary mass, pancreatic and bile duct dilatation	Tumor at paiplla	Surgical sample	Left	Metachronous-8 months	PD	Dead at 14 months	-	No complication
Popovic, 2018 [50]	M, 58 years	Occlusion-bleeding	Anemia		Third duodenum-obstructive mass	Endoscopic biopsy	Left	Metachronous-7 months	Endoscopic thermocoagulation	Dead at 4 years	-	
Nakamura, 2019 [51]	M, 74 years	Vomiting, orthostatic fainting	Severe anemia	EUS: biliary duct dilatation and hypoechogenic mass		EUS biopsy	Right	Metachronous	-	-	-	No complication
Munir, 2019 [52]	M, 76 years	Asymptomatic-diagnosis at following	-	Mass	Second duodenum-friable mass	Endoscopic biopsy	Right	Metachronous-2 years	Sunitinib-nivolumab	-	-	
Farrokh, 2019 [53]	M, 62 years	Fatigue, weigh loss, massive gastrointestinal bleeding		Mass in nephrectomy bed invading D2 and D3	Second duodenum-irregular ulcerative mass	Endoscopic biopsy	Right	Metachronous-10 years	Embolization-axitinib	-	-	
Baghmar, 2019 [54]	M, 84 years	Fatigue, anemia, diarrhea	Severe anemia	Mass with central necrosis	First/second duodenum junction-ulcerations	Endoscopic biopsy	Right	Metachronous-37 years	Palliative treatment		-	
Jain, 2020 [55]	M, 73 years	Abdominal pain, nausea, vomiting, weight loss, hypertension, abdominal tenderness, anemia	Severe anemia	Lymph nodes in pancreaticoduodenal, para-aortic, precaval regions	Periampullary-ulcer	Endoscopic biopsy	Right	Metachronous-33 years	Immunotherapy	-	LN	-
Peters, 2020 [56]	M, 68 years	Dyspnea, fatigue, anemia	Severe anemia	-	Second duodenum-polyp	Endoscopic biopsy	Right	Metachronous-1.5 years	Embolization-axitinib		Bones, live, LN	

PD: pancreaticoduodenoctomy.

**Table 2:** Characteristics of included case.

Feature		n	Average/Percentage
Male/Female ratio		55	4/1
Average age		55	64 years
Metachronous vs. synchronous		55	
Metachronous	49		89%
DFI (median)		45	7 years
Synchronous	6		11%
RCC side		48	
Bilateral	1		2%
Left	19		40%
Right	28		58%
Symptoms	54	55	98%
Lethargy–general symptoms	22		40%
Occlusion	5		9%
Abdominal pain	10		18%
Bleeding (upper and lower)	36		65%
Upper GI bleeding	12		22%
Lower bleeding (Melena, hematoschezia)	26		47%
Anemia	32		58%
Fever	4		7%
Jaundice-cholestasis	5		9%
Other metastasis site	20	55	36%
Localization of the tumor		44	
Second duodenum	35		80%
Periampullary	12		27%
Angulus	2		5%
First Duodenum	6		14%
Third duodenum	1		2%
Treatment		48	
Surgery alone	23		48%
Medical therapy alone	11		23%
Surgery + medical therapy	5		10%
Paliative RT	3		6%
No treatment	6		13%
Overall Survival-Median			48 months

RT: Radiotherapy; RCC: Renal Cell Carcinoma; DFI: Disease Free Interval; GI: Gastro-Intestinal.

**Table 3:** Features of patients treated with surgery vs. patients treated without surgery.

		Surgery (n = 28)	No Surgery (n = 20)	n	*p*
Age, median		60.5 [55.2; 66.0]	70.0 [59.5; 76.0]	48	0.028
Sex (Male proportion)		22 (79%)	16 (80%)	38	1
DFI, median		8.00 [6.00; 11.0]	4.00 [1.50; 9.25]	41	0.067
Bleeding/anemia		22 (79%)	15 (75%)	37	1
Localisation	D2	16 (80%)	14 (74%)	30	0.35
	D1	4 (20%)	2 (11%)	6	
	ANGULUS	0 (0%)	2 (11%)	2	
	D3	0 (0%)	1 (5.3%)	1	
Metachronous metastasis		25 (89%)	17 (85%)	42	0.68
Nephrectomy side	Right	16 (57%)	10 (50%)	26	0.88
	Left	9 (32%)	9 (45%)	18	
	Bilateral	1 (3.6%)	0 (0%)	1	
Other metastasis localisation, n		18 (64%)	15 (75%)	33	0.43

## Data Availability

Not applicable.
